# Why Do Individuals Seek Information? A Selectionist Perspective

**DOI:** 10.3389/fpsyg.2021.684544

**Published:** 2021-11-19

**Authors:** Matthias Borgstede

**Affiliations:** Foundations of Education, University of Bamberg, Bamberg, Germany

**Keywords:** behavioral selection, natural selection, information theory, Fisher information, entropy, multilevel model of behavioral selection, covariance based law of effect, free energy principle

## Abstract

Several authors have proposed that mechanisms of adaptive behavior, and reinforcement learning in particular, can be explained by an innate tendency of individuals to seek information about the local environment. In this article, I argue that these approaches adhere to an essentialist view of learning that avoids the question why information seeking should be favorable in the first place. I propose a selectionist account of adaptive behavior that explains why individuals behave as if they had a tendency to seek information without resorting to essentialist explanations. I develop my argument using a formal selectionist framework for adaptive behavior, the multilevel model of behavioral selection (MLBS). The MLBS has been introduced recently as a formal theory of behavioral selection that links reinforcement learning to natural selection within a single unified model. I show that the MLBS implies an average gain in information about the availability of reinforcement. Formally, this means that behavior reaches an equilibrium state, if and only if the Fisher information of the conditional probability of reinforcement is maximized. This coincides with a reduction in the randomness of the expected environmental feedback as captured by the information theoretic concept of expected surprise (i.e., entropy). *The main result is that behavioral selection maximizes the information about the expected fitness consequences of behavior, which, in turn, minimizes average surprise.* In contrast to existing attempts to link adaptive behavior to information theoretic concepts (e.g., the *free energy principle*), neither information gain nor surprise minimization is treated as a first principle. Instead, the result is formally deduced from the MLBS and therefore constitutes a mathematical property of the more general principle of behavioral selection. Thus, if reinforcement learning is understood as a selection process, there is no need to assume an active agent with an innate tendency to seek information or minimize surprise. Instead, information gain and surprise minimization emerge naturally because it lies in the very nature of selection to produce order from randomness.

## Introduction

Many species adapt their behavior to changing environments by mechanisms of learning. While early psychological learning theories stressed the importance of temporal contiguity between a behavior and its reinforcing consequences (Hull et al., 1940; Thorndike, 2010/1911), more recent approaches often rely on the concept of *prediction*. In this view, stimuli acquire control over behavior only if they are reliable and non-redundant predictors of reinforcers (Egger and Miller, 1962, 1963; Kamin, 1969; Rescorla, 1988; Williams, 1999). The importance of predictiveness for learning seems to imply that individual learning is inherently linked to the *information* that stimuli yield about the expected consequences of behavior (Berlyne, 1957).

Several researchers have incorporated concepts from information theory into theoretical accounts of learning and reinforcement (Hendry, 1965, 1969; Bloomfield, 1972; Ward et al., 2012, 2013). Information theory has also been applied in cognitive accounts of learning and perception (Bubic et al., 2010; Clark, 2013; Gottlieb et al., 2013) and neural theories of adaptive behavior (Friston et al., 2006; Niv, 2009; White et al., 2019). Although these approaches vary considerably in the way that learning is linked to information theory, they all build on the idea that adaptive behavior can be explained by information theoretic concepts. In other words, the tendency to seek information to predict the environment is generally taken to be a first principle, an unexplained explainer. Hence, existing accounts avoid the question why individuals accumulate information about their environment by stipulating an innate tendency to seek information. For example, in the free energy formulation of predictive coding (also known as the *free energy principle*, FEP), adaptive behavior and learning are explained by a tendency to minimize predictive error—a property that is assumed to be constitutive of all living organisms (Friston et al., 2006).

The problem with this line of reasoning is that it adheres to an *essentialist* philosophy of science (Palmer and Donahoe, 1992). Essentialism goes back to Aristotle who held that all phenomena in nature reflect some universal, enduring qualities that are intrinsic to each class or unit. In this view, all categories are defined by essential properties. For example, the category of “red things” is defined as those objects that possess the abstract property of “redness.” Essentialist explanations rely on one or more of such properties that are assumed to be innate to the objects under consideration. For example, Ptolemy’s theory of *epicycles* explained the movement of celestial bodies by an innate tendency to move in circles (Hanson, 1960). Similarly, the theory of *orthogenesis* explained evolutionary change by an innate tendency toward higher levels of organization and complexity (Ulett, 2014).

In biology, essentialism was eventually replaced by Darwin’s theory of evolution by natural selection, which provides a non-teleological account of biological adaptations that does not rely on innate tendencies as explanatory modes. Selectionism also explains the observed orderliness of planetary movement as a by-product of gravitation, since most objects in the solar system either collapse into the sun or leave the system altogether. Whereas essentialism has long been abandoned as “unscientific” in the natural sciences, it still prevails as an explanatory mode in many areas of psychology (Donahoe et al., 1993).

In this paper, I develop a selectionist explanation for the observed connection between learning and information that does not rely on essentialist concepts like an innate tendency to seek information. I use a formal model of behavioral selection that builds on an extended Price equation (Price, 1970, 1972). Although originally intended to describe natural selection on a genetic level, the Price equation has been adapted to explain selection processes in other domains such as cultural selection (Lehtonen, 2020) and individual learning (Baum, 2017). In the context of natural selection, the Price equation has also been applied to information theoretic concepts (Frank, 2017, 2020). In particular, it has been shown that natural selection maximizes the Fisher information of a random observation from a population with regard to the amount of change from the parent population to the descendant population (Frank, 2009).

By analyzing natural selection in terms of Fisher information, Frank (2009) establishes a formal link between the concepts of selection and statistical prediction. However, it is difficult to give an intuitive interpretation to the concept of statistical prediction on the level of an entire population. In contrast to natural selection, when reinforcement learning is interpreted as a selection process, the implied connection between the Price equation and statistical predictiveness seems more intuitive. It is straightforward to conceptualize individuals as learning agents acting according to their statistical predictions about the environment. Therefore, if reinforcement learning is a selection process, a formal link between the Price equation and Fisher information might explain why learning individuals seem to seek information about their environment. The aim of this article is to establish such a formal link.

In the following section I first provide a brief introduction to the multilevel model of behavioral selection (MLBS). The MLBS is based on an extended Price equation that captures behavioral change due to reinforcement learning and evolution simultaneously (Borgstede and Eggert, 2021). I then show that the fundamental principle of behavioral selection, the covariance based law of effect, implies that reinforcement learning coincides with an increase in the information an individual accumulates about the expected fitness consequences of its behavior (section “Behavioral Selection and Fisher Information”). The main result is that behavioral allocation is at equilibrium, if and only if the distribution of expected evolutionary fitness has maximal Fisher information with regard to the consequences of an individual’s average behavior. I further show that this coincides with the individual minimizing average surprise, i.e., information entropy, (section “Relation to Self Information and Entropy”). This latter result suggests a connection between behavioral selection and the *free energy principle* (FEP) proposed by Friston et al. (2006), which is claimed to provide a general theory of adaptive behavior by means of predictive brain processes (Friston, 2010; Badcock et al., 2019). I establish a formal connection between the MLBS and the FEP, thereby showing that both theories arrive at the same predictions from very different assumptions (section “Relation to Predictive Coding”). Whereas the FEP presumes that minimizing surprise explains behavioral adaptations, the MLBS implies that minimizing surprise is a consequence of behavioral selection. Finally, the implications of the results are summarized and discussed (section “Discussion”).

Since the link between learning and information follows directly from the MLBS, there is no need to assume an innate tendency of learning individuals to seek information about their environment. Instead, reinforcement learning coincides with a reduction in randomness (and thus a gain in information) because it lies in the very nature of selection processes to produce order from randomness. This clarifies the role of information theory for reinforcement learning and explains why individuals seem to have a tendency to seek information about their environment.

## The Multilevel Model of Behavioral Selection

It is a long-held belief that reinforcement learning shapes individual behavior in a similar way that natural selection shapes the characteristics of a species (Thorndike, 1900; Pringle, 1951; Skinner, 1966, 1981; Staddon and Simmelhag, 1971). Several attempts have been made to formalize the idea that learning can be understood as a certain type of selection that is often called *behavioral selection* (Donahoe et al., 1993; McDowell, 2004, 2013; Donahoe, 2011; Baum, 2012, 2017). However, these approaches rely on a formal analogy between the mechanisms of learning and the principle of natural selection, thereby missing the opportunity to give a functional integration of learning and evolution. A second line of reasoning conceives learning mechanisms as a result of natural selection (McNamara and Houston, 2009; Singh et al., 2010). However, in these latter approaches, reinforcement learning itself is not described as a selection process. Therefore, until recently, there was no overarching theory that unifies behavioral selection and natural selection in a way that functional relations between both levels of selection can be established.

The MLBS provides such a unifying account (Borgstede and Eggert, 2021). Starting with the most general description of natural selection as the result of traits co-varying with evolutionary fitness as specified in the Price equation (Price, 1970, 1972), the MLBS provides a coherent formal integration of learning and evolution. The core concept is that reinforcers are essentially (context-specific) statistical fitness predictors. Any trait that predicts evolutionary fitness^1^ on the level of the population will naturally function as a reinforcer if it can be changed by individual behavior. Given behavior varies within individuals, this functional relation between (context-specific) behavior and a trait that predicts evolutionary fitness implies a within-individuals covariance between the behavior and the fitness predictor. Any mechanism that fosters behavioral change in the direction of this (individual level) covariance will, on average, yield a higher individual fitness and is thus favored by natural selection. Consequently, the observed similarity between learning and evolution arises because reinforcement learning and natural selection can both be described by means of the covariance principle given by a multilevel extension of the Price equation.

The MLBS relies on a molar conceptualization of reinforcement (Rachlin, 1978). The basic assumptions of this approach are that behavior is inherently variable and extended in time. This implies that behavior is best analyzed on an aggregate level that averages responses emitted in a certain context over time (Baum, 2002). Instead of focusing on single instances of behavior, the molar approach describes patterns of behavioral allocation over time by means of quantitative regularities like, for example, the matching law (Baum, 1974; Herrnstein, 1974). Following this rationale, the MLBS models behavior as the time an individual engages in an activity within a specified context. A context is defined by recurring contingency structures in an individual’s environment. Within this conceptual framework, behavioral change due to reinforcement is analyzed by comparing average behavioral allocation over time between multiple sets of reinforcement trials (so-called *behavioral episodes*).

On the level of the whole population, change in mean behavioral allocation $△\,\bar{b}$ can be expressed by the Price equation (Price, 1970):


(1) $$\bar{w}\,△\,\bar{b}={\text{Cov}}_{i}\,\left(w_{i},b_{i}\right)+{\text{E}}_{i}\,\left(w_{i}\,△\,b_{i}\right)$$

Here, *w*_*i*_ refers to the contribution of individual *i* to the future population (i.e., individual fitness) and $\bar{w}$ designates the corresponding population average in evolutionary fitness. Behavior *b*_*i*_ is conceptualized as the average behavioral allocation of individual *i* in a specified context when averaged over all instances of the corresponding contingency structure. Due to the definition of behavior as time spent engaging in an activity, all *b*_*i*_ are real numbers ranging between zero and the duration of a behavioral episode. Equation (1) separates population change into a covariance term Cov_*i*_ (*w*_*i*_, *b*_*i*_), capturing the effects of natural selection, and an expectation term E_*i*_(*w*_*i*_△*b*_*i*_), capturing the effects of within-individual change. Because the Price equation holds irrespective of the specific mechanisms of transmission, it does not matter here whether the behavioral trait *b* is passed on to the next generation via genetic inheritance or via cultural transmission (e.g., imitation or instruction).

It is possible to expand the Price equation by further separating the fitness weighted within-individual change *w*_*i*_△*b*_*i*_ using the same scheme. Hence, in the MLBS, change within individuals is further partitioned into an individual-level covariance between behavioral allocation and fitness ranging over behavioral episodes *j*, and an individual-level expectation term capturing all sources of within-individual behavioral change that are not selection. The corresponding multilevel Price equation is:


(2) $$\bar{w}\,△\,\bar{b}={\text{Cov}}_{i}\,\left(w_{i},b_{i}\right)+{\text{E}}_{i}\,\left({\text{Cov}}_{j}\,\left(w_{i\,j},b_{i\,j}\right)+{\text{E}}_{j}\,\left(w_{i\,j}\,\Delta \,b_{i\,j}\right)\right)$$

Since fitness is measured as the contribution of an individual to the future population (i.e., fitness is a characteristic of the whole individual), it is not reasonable to ascribe fitness to single behavioral episodes within individuals (as implied by the term *w*_*ij*_). Instead, the MLBS incorporates the concept of context-dependent fitness predictors by means of a linear regression that predicts individual fitness on the population level using a fitness predictor *p* with *w* = β_0_ + β_*wp*_*p* + ε. Substituting the *w*_*ij*_ with the corresponding predicted values, it is possible to describe the individual change in behavioral allocation for each individual by the following equation:


(3) $$w_{i}\,△\,b_{i}=\beta_{w\,p}\,{\text{Cov}}_{j}\,\left(p_{i\,j},b_{i\,j}\right)+\delta$$

Equation (3) gives an abstract description of reinforcement learning by means of behavioral selection and is called the *covariance based law of effect:* the change in behavior due to behavioral selection is proportional to the covariance between behavior and a fitness predictor, and proportional to the statistical effect of the fitness predictor on evolutionary fitness. The term Cov_*j*_ (*p*_*ij*_, *b*_*ij*_) refers to the within-individual covariance between behavior *b*_*ij*_ and reinforcer *p*_*ij*_ over several behavioral episodes (e.g., trials in a behavioral experiment). The residual term δ captures all influences on behavioral change that are not selection. β_*wp*_ is the slope of the regression of evolutionary fitness on reinforcement (also referred to as *reinforcing power*) and may be different in various contexts.^2^

The covariance based law of effect is closely related to the concept of *reinforcer value* as a behavioral maximand. The idea that individuals behave as if they were maximizing some quantitative measure of value is a common theme in behavioral psychology (Rachlin et al., 1981), behavioral economics (Rachlin et al., 1976), behavioral ecology (Davies et al., 2012) and formal accounts of reinforcement learning (Frankenhuis et al., 2019). However, few have attempted to explore the formal constraints on reinforcer value from an evolutionary perspective (McNamara and Houston, 1986; Singh et al., 2010). Since the behavioral outcome of reinforcement affects individual fitness, the values assigned to different behaviors cannot be independent of natural selection. It has been shown that, if reinforcer value and evolutionary fitness are maximized simultaneously, marginal reinforcer value *r*(*b*) coincides with the expected gain in evolutionary fitness per unit change in behavioral allocation (Borgstede, 2020).

**FIGURE 1:**
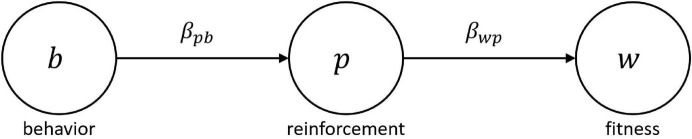
Path model relating behavior b to evolutionary fitness w via reinforcement p. Following the MLBS, any statistical fitness predictor p will act as a reinforcer. The partial regression coefficients β_pb_ and β_wp_ designate the slope of the environmental feedback function relating behavior to reinforcement, and the slope of the fitness function relating reinforcement to fitness, respectively. The product of both partial effects constitutes the total effect of behavior on evolutionary fitness, which is equivalent to the reinforcer value r(b) of the behavior (see Borgstede, 2020 for details).

In the context of the MLBS, this expected gain in evolutionary fitness can be retrieved from the covariance based law of effect. Given behavior affects fitness only by means of changes in reinforcement, reinforcer value can be expressed in terms of a statistical path model where the effect of behavior *b* on evolutionary fitness *w* is completely mediated by reinforcement *p* (see Figure 1). Consequently, the total fitness effect of *b* equals the product of the partial regression effects β_*pb*_ and β_*wp*_, and marginal reinforcer value becomes *r*(*b*) = β_*wp*_β_*pb*_. Since, by standard covariance calculations, β_*pb*_ = Cov_*j*_ (*p*_*ij*_, *b*_*ij*_)/Var(*b*_*ij*_), Equation (3) can be rearranged to:


(4) $$w_{i}\,△\,b_{i}=r\,\left(b_{i}\right)\,\text{Var}\,\left(b_{i\,j}\right)+\delta$$

Thus, the covariance based law of effect implies that behavioral change due to reinforcement is proportional to marginal reinforcer value and proportional to the intra-individual behavioral variance. If the fitness function of *b* is a smooth concave function with a global maximum, behavioral selection will foster change until there is no further gain in reinforcer value (i.e., *r* (*b*) = 0). This implies that absolute reinforcer value (in terms of evolutionary fitness) is maximized.

**FIGURE 2:**
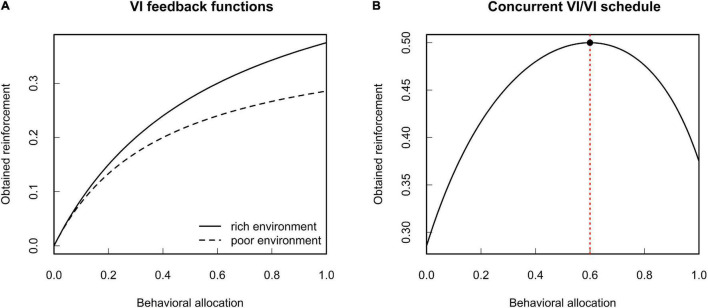
Expected environmental feedback from variable interval (VI) schedules of reinforcement. **(A)** Two VI feedback functions for different average time between reinforcements. **(B)** Total expected reinforcement obtained from a concurrent VI/VI schedule of reinforcement. The red dotted line indicates the point where the marginal gain in reinforcement is zero. Given constant fitness functions, this coincides with the maximum amount of obtained reinforcement in terms of absolute reinforcer value (see Borgstede, 2020 for details).

The MLBS gives a formal account of reinforcement learning by means of an abstract selection principle. Its import for the theoretical analysis of behavior is best demonstrated by an example. Consider an organism that adapts its foraging behavior to the current environment by means of behavioral selection (i.e., reinforcement as specified by the MLBS). Let us assume that, in a given environment, there are two food patches. Given equal foraging effort, the average time to encounter food varies between the two patches. Restricting ourselves to the exploitation of these two food sources, behavioral allocation *b* can be expressed by a single number referring to the time spent at one of the food patches (the time spent at the other food patch is given implicitly by the duration of a behavioral episode). Given food is not constantly available at the patches, the animal is subject to a concurrent variable interval (VI) schedule of reinforcement. With regard to the amount of reinforcement obtained from both options, variable interval schedules yield a concave feedback function that depends on the relative reinforcement rates. Figure 2 illustrates two such feedback functions, along with the total amount of expected reinforcement.^3^ Imagine the animal is exposed to this contingency repeatedly. At each trial, the individual will slightly vary its own behavioral allocation. Since reinforcement is contingent on behavior, this will result in a corresponding variation in reinforcement between the trials. On an aggregate level, this contingency can be expressed in terms of the covariance between behavioral allocation and reinforcement. The covariance based law of effect states that behavior changes in the direction of this covariance, with the rate of change depending on the expected gain in evolutionary fitness per unit change in reinforcement. From the shape of the feedback function it follows that this covariance will be zero, if and only if behavioral allocation is chosen such that it maximizes the sum of reinforcement received from the two patches (compare Figure 2B). Given that both patches yield the same food (and hence identical fitness effects per unit of reinforcement), maximization of reinforcement coincides with the well-known matching law (Baum, 1981).

## Behavioral Selection and Fisher Information

Fisher information measures the amount of information an observation provides with regard to an unknown parameter of the underlying probability distribution. Fisher information is mostly used in statistical estimation theory. In the context of behavioral selection, the question arises whether the statistical regressions stipulated by the MLBS can be exploited to mimic an agent based approach to reinforcement learning where the individual adapts its behavior by predicting the expected (fitness) consequences of its own behavior. If such an interpretation is possible, we would expect the individual to act as if it was constructing statistical estimates about its expected fitness. In this case, the information of a random observation (i.e., the environmental feedback to the individual’s behavior) with regard to the true individual fitness would be captured by the corresponding Fisher information.

To give a general definition of Fisher information, suppose a random variable *X* that is characterized by a probability distribution function with a given parameter θ. Let the likelihood of this parameter with regard to *X* be designated by *L*_*X*_(θ). Fisher information is defined as the variance of the first derivative of the log-likelihood with regard to the underlying parameter:


(5) $$F_{X}\,\left(\theta \right)=\text{Var}\,\left(\frac{d\,\text{log}\,\left(L_{x}\,\left(\theta \right)\right)}{d\,\theta }\right)$$

Given the underlying distribution satisfies certain regularity conditions, this is equivalent to the curvature of the log-likelihood in the region of the maximum (Lehmann and Casella, 1998). For example, if *X* is a normally distributed variable with given variance σ^2^, the Fisher information of the expected value μ is:


(6) $$F_{X}\,\left(\mu \right)=\text{Var}\,\left(\frac{d\,\text{log}\,\left(L_{x}\,\left(\mu \right)\right)}{d\,\mu }\right)=\frac{1}{\sigma^{2}}$$

Thus, for a normally distributed random variable, a small variance yields a high Fisher information. In other words, the smaller the variance, the more information about the expected value can be obtained from a random observation.

Since in the MLBS behavioral change only occurs to the degree to which reinforcement predicts evolutionary fitness, it is plausible that individuals should behave in such a way that they can reliably predict the fitness consequences of reinforcement. Formally, individuals are expected to behave such that the environmental feedback has minimum variance and hence yields maximum Fisher information. In the following, I will show that this tendency to maximize Fischer information naturally arises when reinforcement is understood as a selection process.

In the MLBS, expected evolutionary fitness is treated as a probabilistic function of average behavior. Equation (4) states that change in average behavior is proportional to marginal reinforcer value *r* (*b*), which corresponds to the expected change in evolutionary fitness per unit change in behavior. As before, we assume that behavior is inherently variable, and that *r* (*b*) is given by the slope of a linear regression of evolutionary fitness on individual behavior with the standard assumption of a normally distributed random error of constant variance. We further assume a smooth concave fitness function with a global maximum, as it is naturally produced by environmental contingencies with diminishing returns (like the abovementioned VI/VI schedules of reinforcement).

Under these assumptions, the conditioned probability of individual fitness is a normally distributed random variable *W*_*i*_ with expectation μ_*i*_ = *w*_*i*_ and variance $\sigma_{i}^{2}=\text{Var}\,\left(w_{i\,j}\right)$. A random observation from *W*_*i*_ corresponds to the environmental feedback conditioned on average behavior^4^ (i.e., the reinforcing consequences of the behavior in terms of expected evolutionary fitness). By Equation (6) the Fisher information of a random observation from *W*_*i*_ with regard to expected fitness μ_*i*_ is:


(7) $$F_{W_{i}}\,\left(\mu_{i}\right)=\frac{1}{\text{Var}\,\left(w_{i\,j}\right)}$$

**FIGURE 3:**
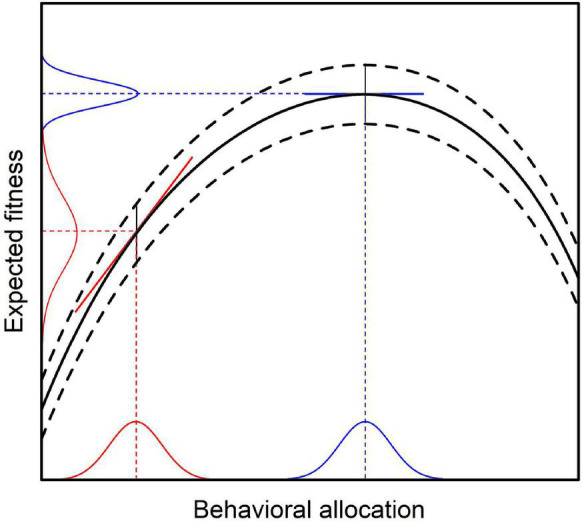
Relation between average behavior and variance in expected evolutionary fitness as implied by the MLBS. Given constant error variance (indicated by the curved dashed lines), the variance of the expected fitness consequences conditioned on average behavior depends on behavioral variance and the slope of the fitness function (i.e., marginal reinforcer value). Consequently, the variance of expected fitness will be higher than the error variance when the slope of the fitness function has a high absolute value (red lines). The covariance based law of effect predicts that behavioral change is proportional to marginal reinforcer value. Hence, when the slope of the fitness function is zero (blue lines), there will be no change in average behavior. This coincides with the point where the variance in expected fitness is smallest, resulting in maximum Fisher information about the expected fitness consequences conditioned on average behavior.

From Equation (7) it follows immediately, that Fisher information with regard to expected evolutionary fitness reaches its maximum, if and only if Var(*w*_*ij*_) is as small as possible. Given the above assumptions, behavioral selection will eventually change behavior toward the point of maximum expected evolutionary fitness (Borgstede, 2020). At this point, marginal reinforcer value *r*(*b*_*i*_) will be zero and behavioral selection will cease (compare Figure 2B). With decreasing marginal reinforcer value, Var(*w*_*ij*_) will also decrease until it reaches its minimum value at the point of behavioral equilibrium. This conclusion follows directly from the relation between the slope of the fitness function and the variance in expected evolutionary fitness as illustrated in Figure 3 (see Appendix for a formal derivation).

The individual can thus be conceptualized to adjust its behavior to the environment, such that the information obtained from the behavioral consequences with regard to expected evolutionary fitness is maximized.

## Relation to Self-Information and Entropy

In the context of behavioral selection, Fisher information is closely related to another information theoretic concept, known as Shannon information or *entropy*. Shannon information was originally used to quantify the information content of a message that is transmitted from a sender to a receiver (Shannon, 1948). In a broader sense, it provides a nonparametric measure of the randomness of a probability distribution. In other words, with higher entropy it becomes more difficult to make valid predictions. Therefore, if individuals adjust their behavior according to their predictions about their expected evolutionary fitness, predictions are reliable if and only if the entropy of the conditional fitness distribution is low. Thus, when behavior is adjusted by means of behavioral selection, a low entropy should be more favorable than a high entropy.

Formally, entropy is defined by means of self-information or *surprise*. Given a probability distribution *X*, the self-information *I* (*x*) of an event *x* equals the negative logarithm of its associated probability *P* (*x*):


(8) I⁢(x)=-log⁢(P⁢(x))

The expected value of self-information (i.e., the average surprise) for the whole distribution is called Shannon information, or entropy *H*(*X*):


(9) $$H\,\left(X\right)={\text{E}}_{i}\,\left(I\,\left(x_{i}\right)\right)$$

For a normally distributed random variable *X* with variance σ^2^, this corresponds to:


(10) $$H\,\left(X\right)=\frac{1}{2}\,\log\left(2\,\pi \,e\,\sigma^{2}\right)$$

Because all terms inside the logarithm in Equation (10) apart from σ^2^ are positive constants, the entropy of a normally distributed random variable is a monotone increasing function of its variance σ^2^. Since the Fisher information for a normal distribution is given by $\frac{1}{\sigma^{2}}$, maximizing Fisher information minimizes entropy in this case. Therefore, behavioral selection leads to minimizing the randomness of the environment with regard to predicted fitness in a given environment. Learning can thus be understood as a process that maximizes the information about expected evolutionary fitness and minimizes the average surprise (i.e., entropy) associated with the consequences of behavior.

## Relation to Predictive Coding

The above calculations show that, in the MLBS framework, individuals are expected to behave as if they were maximizing the Fisher information about their expected evolutionary fitness or, equivalently, as if they were minimizing average surprise obtained from environmental feedback with regard to their expected individual fitness. These results follow straightforward from the covariance based law of effect, which provides a strictly behavioral account of reinforcement on a molar level of analysis.

In this section, I will explore the theoretical implications of these results with regard to the *theory of predictive coding* (TPC), where the individual is conceived as an active agent that adapts its behavior by means of an innate tendency to minimize predictive error between perception and its current expectations (Clark, 2013). Predictive coding is chosen as a case study here because it can be regarded as a paradigmatic example of an essentialist mode of explanation in psychology. The TPC is a neuro-cognitive account of adaptive behavior that builds on the concept of agency. This means that individuals are conceived as active decision makers that form internal representations of the environment (generative models) and use the information they obtain from environmental feedback to update their internal world model and their current behavior. In the predictive coding framework, perception and action are understood as being the simultaneous result of a continuous process that minimizes predictive error.

On a conceptual level, the idea of an active agent continuously seeking to minimize predictive error seems to contradict the behavioral view expressed in the MLBS. The first obvious difference concerns the question what counts as behavior (or “action” in the predictive coding terminology). The TPC describes behavior as a continuous stream of action and thus provides a molecular perspective to adaptive behavior. The MLBS describes behavior on a different level of analysis. Instead of describing every single action in a continuous stream of behavior, the MLBS focuses on average behavior that is itself extended over time. This corresponds to the aforementioned molar view (Baum, 2002, 2013). The difference between molecular and molar theories of behavior is analogous to the difference between classical mechanics, which describes the motion of single particles, and statistical mechanics, which describes the same particles on an aggregate level. It makes little sense to say that one of the approaches is superior to the other as such. When dealing with a comparably simple system within a limited time frame, a molecular analysis may be the best choice. However, when the system becomes more complex or the time scale becomes more extended, molecular analyses often fail to produce accurate predictions (this also holds for physical systems). Therefore, in these cases a molar level of analysis can provide a better picture. Nevertheless, both approaches deal with the same kind of phenomena—hence the results of a molecular model should, in theory, coincide with a corresponding molar model. Therefore, if the implied connection between the MLBS and the TPC is supposed to be more than metaphorical, the above results should be consistent with the general framework of the TPC.

The second difference between the MLBS and the TPC is a matter of perspective. The TPC describes behavior from the perspective of the individual. This means that individuals and their representations of the world are the primary object of study. Consequently, the principle of error minimization is formulated such that it can be applied to individual agents that actively seek information and choose their corresponding actions such that they fit the perceived environment best. The MLBS describes adaptive behavior from the perspective of the environment. This means that the contingencies in the environment are the primary focus of the analysis. Hence, the MLBS does not invoke internal representations or innate tendencies of the individual. Instead, adaptive behavior is described as an environmental selection process that changes the state of the individual. The TPC gives an essentialist account of information seeking, allocating the source of change inside the object (in the form of innate powers), whereas the MLBS gives a selectionist account, allocating the source of change outside the object (in the form of applied forces). Whilst the latter approach has become the predominant philosophy of modern natural science, it is still a point of debate whether it is suitable to describe the behavior of living organisms. Therefore, if the MLBS can give a coherent explanation for the apparent tendency of individuals to strive for better predictions without invoking an essentialist mode of explanation, this would be a strong case against the necessity of innate powers to explain behavior.

In the following, both issues shall be addressed in order to clarify the theoretical implications of the above analysis. I focus on a formalized version of predictive coding known as the *free energy principle* (FEP) that was introduced by Friston et al. (2006) and has been applied to model adaptive behavior in several domains, like perception, motor control, optimal choice and neural plasticity (see Friston, 2010 for a review). In the following, I adhere to the formalism presented in Buckley et al. (2017) due to its notational simplicity and clarity of presentation.^5^

In the FEP framework, the concept of predictive error minimization is conceptualized by means of an agent that forms an internal representation of the environment (a *generative model*) and uses this model to predict which sensory states should occur in the future. The *actual* sensory states are the result of the environmental feedback to the individual’s actions and may depart from these predictions. The first source of prediction error lies in the stochasticity of the environmental feedback. The second source arises when the generative model differs from the actual contingencies of the environment (i.e., the individual’s internal representation of the structure of the world is flawed). The core assumption of the FEP is that both, the parameters of the generative model and the actions of the individual, are chosen such that the actual sensory states (i.e., the experienced environmental feedback) are most likely.

Formally, the environment is characterized by the values of a set of environmental variables (which are called *environmental states*) that jointly affect the values of a set of internal variables (which are called *sensory states*). The state of the world shall be designated ϑ, the sensory states φ. The probability density over sensory states (given the individual’s generative model) is designated *p*(φ) and the probability density over environmental states, given sensory states *p*(*ϑ*|φ), respectively.

In the FEP, it is assumed that the true state of the world is not accessible to the individual and, consequently, the conditional probability *p*(*ϑ*|φ) cannot be calculated exactly but has to be approximated by the individual’s “best guess,” the *recognition density q*(*ϑ*). The divergence between the true probability density over environmental states and the individual’s recognition density introduces an additional source of predictive error, which is formally captured by the Kullback-Leibler divergence^6^ *D*(*q*(*ϑ*)||*p*(*ϑ*|φ)). The sum of this Kullback-Leibler divergence and the self-information of the sensory states −log⁡(*p*(φ)) is called *free energy F* (due to its formal similarity to the concept of free energy from statistical mechanics):


(11) F=-log(p(φ))+D(q(ϑ)||p(ϑ|φ))

The free energy principle now states that all parts of the behavioral system that can change (i.e., the parameters of the generative model and the action states of the individual) are chosen by the individual such that free energy *F* is minimized.

To investigate the relation between the MLBS and the FEP, we need to provide a free energy formulation of the kind of behavioral system that is studied by molar theories of reinforcement. We only consider the case of constant reinforcing power β_*wp*_ (i.e., the individual’s sensitivity to reinforcement is the same in every behavioral episode). Under this condition, the amount of reinforcement and the expected evolutionary fitness only differ by a constant factor. Hence, maximum expected fitness coincides with maximum reinforcement and Equation (7) can be equivalently stated for the conditional distribution of expected reinforcement. Consequently, given constant reinforcing power, behavioral selection does not only maximize Fisher information with respect to expected evolutionary fitness, but also with respect to expected reinforcement. Because Fisher information is inversely related to information entropy, the MLBS predicts that average surprise (i.e., entropy) with regard to expected reinforcement will be minimized.^7^

A free energy formulation for simple scenarios like the above foraging example is straightforward. Here, the true structure of the world is given by the feedback functions associated with the food patches. We can thus identify the environmental states ϑ with the slopes of the feedback functions. The sensory states φ are a direct consequence of the amount of food that the individual actually receives and may consist in smelling or tasting the food obtained from the two patches. Since smelling or tasting food usually predicts higher evolutionary fitness, the sensory states are reinforcers in the sense of the MLBS. Hence, *p*(*φ*) is the probability of reinforcement, *p*(*ϑ*|φ) is the true probability of the slopes of the feedback functions given reinforcement (which is not known to the individual) and *q*(*ϑ*) is the individual’s “best guess” about the slopes of the feedback functions.

Let us, for the sake of simplicity, assume that the individual has optimized its generative model such that the recognition density *q*(*ϑ*) approximates the true probability density *p*(*ϑ*|φ) as closely as possible (i.e., the individual cannot further reduce the Kullback-Leibler divergence *D* by updating its generative model). In this case, *D* can be treated as a constant and minimization of free energy *F* coincides with minimization of −log(*p*(φ)), which, in the reinforcement scenario, is the surprise (or self-information) of a random observation with regard to expected reinforcement. Consequently, when averaged over a longer period of time, the free energy formulation states that average surprise (i.e., entropy) with regard to expected reinforcement is minimized. Therefore, in the above example, there is a direct correspondence between the molar predictions of the MLBS and the molecular mechanisms of the FEP.

## Discussion

In this paper, I approached the question why reinforcement learning leads to information gain from a selectionist point of view. I provided a formal argument that builds on a re-interpretation of behavioral selection from an information theoretic perspective. It was shown that the covariance based law of effect (as specified in the MLBS) can be formally linked to an agent based approach to reinforcement, where the individual adapts its behavior to the environment by predicting the expected consequences of its own behavior. In this interpretation, individuals adapt their behavior to the environment such that the Fisher information with regard to expected individual fitness is maximized. This coincides with individuals behaving as if minimizing the average surprise (i.e., information entropy) associated with the environmental feedback to their behavior. I further demonstrated that the selectionist account provides an explanation for the observed tendency of individuals to seek information by relating the MLBS to a formalized version of the theory of predictive coding (the free energy principle, FEP). In the FEP, information seeking is stipulated as an essential property of living organisms without further explanation. In contrast to essentialist explanations of adaptive behavior, the selectionist account put forward in this paper demonstrates that information gain emerges from reinforcement being a selection process. Consequently, individuals do not actually seek information. They just appear to do so because their behavior changes as a result of a selection process.

The main import of the MLBS for understanding the relation between learning and information gain is that we do not need to invoke essentialist explanations of adaptive behavior. Selection naturally produces systems that reduce randomness. In biology, selectionism has replaced the historically older view that nature strives toward some ultimate goal (like complexity or perfection). Consequently, the theory of natural selection replaces *teleologic* explanations (“giraffes have a long neck because they need to reach high hanging leaves”) with *teleonomic* explanations (“giraffes have a long neck because reaching high hanging leaves co-varies with evolutionary fitness”). Although seemingly intentional, developing a long neck is no longer seen as the result of an innate tendency or a goal-directed process. In other words, we would not be inclined to say that a species developed a long neck *because it wanted* to reach high hanging leaves.

In contrast to biology, teleologic explanations are still very common in psychology. For example, individuals that approach another person in a bar might be said to do so *because they want to find a partner*. The selectionist account of behavior offers a corresponding teleonomic explanation: individuals that approach another person in a bar do so *because approaching another person co-varies with evolutionary fitness predictors like potential mating opportunities*. If individual changes in behavior can be described by the same abstract principle of selection as population changes in biological traits, we might reconsider how we formulate psychological theories.

The problem with essentialist explanations becomes even more obvious when we consider selection processes outside the realm of biology. For example, the solar system may be regarded as the result of an ongoing selection process (cf. Gehrz et al., 1984). Depending on its velocity and direction of movement relative to the sun, each planet will either collapse into the sun, leave the system or remain in a stable orbit. For a planet to remain in orbit, it needs just the right amount of velocity tangent to its orbit to compensate for the gravitational force that drags it toward the sun. Eventually, the only objects that remain to be observed are the ones that had the requisite velocities. Like all selection processes, the shaping of the solar system is accompanied by an increase in predictability. The particle cloud from which the solar system evolved was a chaotic system. In other words, although each trajectory may be determined by its initial condition, the complex interaction between the particles allow only for probabilistic predictions. However, the selection process outlined above eventually produced highly predictable trajectories. Consequently, within the solar system, we have an increase in information as a direct result of selection.

Imagine we could observe the evolution of the solar system but had no knowledge of the underlying selection process. We might explain the increasing orderliness of movement by means of an innate tendency of solar systems to seek information, just like we explain the increasing orderliness of behavior by means of an innate tendency of individuals to seek information. However, if adaptive behavior is the result of a selection process, we have no reason to accept essentialist explanations of individual behavior any more than we accept an essentialist explanation of the orbits in the solar system.

Behavioral selection theory has made considerable advances in recent years. Whereas the analogy between learning and evolution has been around for over a century, the MLBS states that learning and evolution do indeed follow the same abstract principle of selection. This paper shows that selection can also account for the principle of information maximization—or, equivalently, surprise minimization. Whereas other approaches treat the tendency to minimize predictive error (or surprise) as axiomatic (Friston et al., 2006; Niv, 2009), the MLBS offers an explanation on the level of ultimate (i.e., evolutionary) causes. This means that *learning is not explained by an innate tendency to seek information, but by the nature of selection itself*.

The formal correspondence between selection and information gain supports the view that selection may be understood as a fundamental principle by which nature generates order from randomness and may thus explain why evolution apparently has a tendency to produce increasing levels of complexity and organization (Brooks et al., 1989; Collier, 1998). Behavioral selection theory states that the connection between information theory and selection equally applies to the level of individual learning, thereby offering a conceptual framework for theories of learning and behavior in general that avoids essentialist thinking.

## Author Contributions

The author confirms being the sole contributor of this work and has approved it for publication.

## Conflict of Interest

The author declares that the research was conducted in the absence of any commercial or financial relationships that could be construed as a potential conflict of interest.

## Publisher’s Note

All claims expressed in this article are solely those of the authors and do not necessarily represent those of their affiliated organizations, or those of the publisher, the editors and the reviewers. Any product that may be evaluated in this article, or claim that may be made by its manufacturer, is not guaranteed or endorsed by the publisher.

## Footnotes

1 Following the Price equation framework, in the MLBS, “evolutionary fitness” is understood as the contribution of an individual to the future population with respect to the evolving trait. This contribution does not necessarily coincide with the number of offspring but may also include the probability of an individual’s own survival, as well as the survival of its offspring. Moreover, since behavior can also be transmitted to the future population via non-genetic inheritance mechanisms (like imitation, instruction or cultural artifacts), the Price equation framework can be used to include biologically, as well as culturally mediated fitness effects (El Mouden et al., 2014; Aguilar and Akçay, 2018).

2 For primary reinforcers, like food or physical threat, the fitness gain per unit of reinforcement is most likely coded in the organism’s genome resulting in condition-dependent motivational responses to the corresponding reinforcer. However, knowledge about fitness consequences may also stem from other sources like, for example, observation of other individuals.

3 For an overview of different feedback functions for variable interval schedules of reinforcement see Nevin and Baum (1980).

4 Note that in the MLBS behavior is conceptualized as inherently variable. If behavior were constant over trials, the variance of the expected fitness consequences would also be constant. However, without variation there is no selection, which is why we condition on average behavior with variance Var(*b*_*i**j*_) > 0.

5 Note that there are different versions of the FEP employing different notational conventions, as well as different partitionings of terms.

6 The Kullback-Leibler divergence is an information theoretic measure that quantifies the difference in randomness (as measured by entropy) between a random variable given an estimated model as compared to the randomness given the true model.

7 The assumption of constant reinforcing power is not strictly necessary to connect the MLBS to the FEP. An alternative formulation would treat the conditional distribution of absolute *reinforcer value* (i.e., the integral of *r*(*b*)) as the objective of free energy minimization (cf. Borgstede, 2020). However, from the perspective of the FEP, it may be more intuitive to focus on reinforcement itself because it is often associated with direct sensory inputs like taste or smell.

## Appendix

### Proof That Behavioral Selection Maximizes Fisher Information

Within the MLBS, individuals are assumed to adapt their behavior to the environment using statistical fitness predictors. Changes in behavior are thus linked to expected changes in evolutionary fitness by the covariance based law of effect:

$$w_{i}\,△\,b_{i}=r\,\left(b_{i}\right)\,\text{Var}\,\left(b_{i\,j}\right)+\delta \;\left(A\,1\right)$$

Because marginal reinforcer value *r* (*b*_*i*_) corresponds to the slope of an individual-level regression of fitness on behavioral allocation, we can express the expected fitness values for each behavioral episode by means of an individual predictive model for the fitness associated with each behavior:

$$w_{i\,j}={\hat{w}}_{i\,j}+\varepsilon =\beta_{0\,i}+r\,\left(b_{i}\right)\,b_{i\,j}+\varepsilon \;\left(A\,2\right)$$

Since the expected value of the predicted fitness $\text{E}\,\left({\hat{w}}_{i\,j}\right)$ corresponds to the true individual fitness *w*_*i*_, this model estimates the expected evolutionary fitness of an individual, given the current behavior in the present environment. Given the standard assumptions of linear regression, the (within-individual) variance in expected fitness is:

$$\text{Var}\,\left(w_{i\,j}\right)=\text{Var}\,\left({\hat{w}}_{i\,j}\right)+\text{Var}\,\left(\varepsilon \right)\;\left(A\,3\right)$$

Since the variance of the expected fitness $\text{Var}\,\left({\hat{w}}_{i\,j}\right)$ can be retrieved from the predictive model, it holds that $\text{Var}\,\left({\hat{w}}_{i\,j}\right)=\text{Var}\,\left(\beta_{0\,i}+r\,\left(b_{i}\right)\,b_{i\,j}\right)=r\,{\left(b_{i}\right)}^{2}\,\text{Var}\,\left(b_{i\,j}\right)$. Hence, the variance of the expected evolutionary fitness conditioned on individual behavior is proportional to the squared marginal reinforcer value.

By standard assumptions of linear regression, the conditioned probability of predicted individual fitness is a normally distributed random variable *W*_*i*_ with expectation $\mu_{i}=w_{i}=\text{E}\,\left({\hat{w}}_{i\,j}\right)$ and variance σ^2^ = *r* (*b*_*i*_)^2^Var (*b*_*ij*_) + Var (ε). A random observation from *W*_*i*_ corresponds to the environmental feedback conditioned on average behavior (i.e., the reinforcing consequences of the behavior in terms of expected evolutionary fitness).

The Fisher information of *W*_*i*_ with regard to μ_*i*_ can now be calculated using Equation (7):

$$F_{W_{i}}\,\left(\mu_{i}\right)=\frac{1}{\text{Var}\,\left(w_{i\,j}\right)}=\frac{1}{r\,{\left(b_{i}\right)}^{2}\,\text{Var}\,\left(b_{i\,j}\right)+\text{Var}\,\left(\varepsilon \right)}\;\left(A\,4\right)$$

From Equation (A4) it follows that, if *r*(*b*_*i*_) = 0, Fisher information will be $\frac{1}{\text{Var}\,\left(\varepsilon \right)}$. Given that Var(ε) is constant over the whole range of *b*, this is the maximal possible value of *F*_*W*_*i*__ (μ_*i*_). Therefore, under the given assumptions, behavioral selection maximizes Fisher information.
